# Diagnostic precision of a deep learning algorithm for the classification of non-contrast brain CT reports

**DOI:** 10.3389/fradi.2025.1509377

**Published:** 2025-05-09

**Authors:** Hamza Eren Güzel, Göktuğ Aşcı, Oytun Demirbilek, Tuğçe Doğa Özdemir, Pelin Berfin Erekli

**Affiliations:** 1 Department of Radiology, İzmir City Hospital, İzmir, Türkiye; 2 School of Science and Technology, IE University, Segovia, Spain; 3 School of Management, Technical University of Munich, Munich, Germany

**Keywords:** artificial intelligence, report classification, computed tomography, deep learning, noncontrast head CT

## Abstract

**Objective:**

This study aimed to determine the diagnostic precision of a deep learning algorithm for the classificaiton of non-contrast brain CT reports.

**Methods:**

A total of 1,861 non-contrast brain CT reports were randomly selected, anonymized, and annotated for urgency level by two radiologists, with review by a senior radiologist. The data, encrypted and stored in Excel format, were securely maintained on a university cloud system. Using Python 3.8.16, the reports were classified into four urgency categories: emergency, not emergency but needs timely attention, clinically non-significant and normal. The dataset was split, with 800 reports used for training and 200 for validation. The DistilBERT model, featuring six transformer layers and 66 million trainable parameters, was employed for text classification. Training utilized the Adam optimizer with a learning rate of 2e-5, a batch size of 32, and a dropout rate of 0.1 to prevent overfitting. The model achieved a mean F1 score of 0.85 through 5-fold cross-validation, demonstrating strong performance in categorizing radiology reports.

**Results:**

Of the 1,861 scans, 861 cases were identified as fit for study through the senior radiologist and self-hosted Label Studio interpretations. It was observed that the algorithm achieved a sensitivity of 91% and a specificity of 90% in the measurements made on the test data. The F1 score was measured as 0.89 for the best fold. The algorithm most successfully distinguished emergency results with positive predictive values that were unexpectedly lower than in previously reported studies. Beam hardening artifacts and excessive noise, compromising the quality of CT scan images, were significantly associated with decreased model performance.

**Conclusion:**

The proposed deep learning algorithm demonstrated high diagnostic accuracy, sensitivity, and specificity in classifying non-contrast brain CT reports. These results indicate the feasibility of automated identification of critical cases, which may support workflow efficiency and timely patient management in radiology practice.

## Introduction

In contemporary medical practice, diagnostic imaging is crucial for expeditious and accurate diagnosis, treatment, and monitoring of diverse diseases. However, the misuse of imaging modalities is prevalent, which can result in adverse outcomes, including increased patient exposure to ionizing radiation, elevated healthcare expenditures, and subsequent cascades of additional imaging examinations (1, 2). Global utilization of diagnostic imaging modalities, particularly computed tomography (CT) and magnetic resonance imaging (MRI), has undergone a substantial surge, resulting in an upward trend in the incidence of unjustified, low-value examinations (2, 3). Moreover, it was found that 20%–50% of CT scans in the U.S. are unnecessary (4). This is concerning since CT accounts for less than 10% of all procedures, yet it contributes more than 60% of the total effective dose from imaging examinations (5). To address the overuse of CT scans, several approaches are currently available. These include establishing clear diagnostic imaging guidelines, offering alternative imaging options, involving specialists, conducting post-imaging reviews, and providing feedback by comparing each referrer's ordering habits with the department's average (5). Clinical imaging guidelines have a limited impact on reducing unnecessary scans because of several challenges. These challenges include financial issues, limited equipment availability, workplace culture, lack of awareness of guidelines, fear of missing a diagnosis, self-referrals, patient demands, and, most importantly, poor implementation of the guidelines (1, 6, 7). Regular retrospective audits can help monitor the impact of quality improvement efforts and ensure compliance with guidelines. However, many institutions struggle to perform these audits routinely due to limitations in staffing, funding, and time. Clinical decision support (CDS) systems and more reliable real-time tools for applying standards during routine clinical care could help address these challenges (8, 9).

Two studies demonstrated that automated analysis of radiology referrals is possible by using natural language processing (NLP) to interpret unstructured clinical information, along with machine learning (ML) and deep learning (DL) techniques for classifying referrals as either justified or unjustified. In the first study, this approach was tested and shown to be effective (10), as researchers performed a manual retrospective audit of 375 brain CT referrals using the iGuide system, combining unjustified and potentially justified referrals because of limited data. In a second study, 1,020 lumbar spine MRI referrals from two clinical sites were analyzed for justification, with the data labeled based on clinical expertise (11).

A key aspect of our study was creating an AI-based interpreter for the iGuide system (Quality and Safety in Imaging GmbH, Vienna, Austria) using real-world data. This was done to help apply justification standards more effectively in radiology practice, especially with the increasing use of diagnostic imaging, the ongoing issue of inappropriate CT scans, and the limitations of previous research. Thus, we aimed to determine the diagnostic precision of a selected large language model for the classification of brain CT reports. Such a model poses an alternative way to screen the reports and could help alleviate the problem of unnecessary follow-up CT scans.

## Methods and materials

We first introduce how we gathered and maintained the data, and the labeling process. Next, we discuss the large language model we used and its features.

### Data sourcing and labeling

Randomly selected 1,861 Non-contrast brain CT reports were anonymized and annotated for urgency level by two radiologists and reviewed by one senior radiologist. The research ethics committee at our institution (Ref#161) approved an ethics exemption for the study. Each participating clinical site assessed data privacy concerns and granted an exemption from a full ethical review. The data, which was anonymized, encrypted, and stored in Excel format, was securely kept on a university cloud system. Data analysis was performed using Python version 3.8.16.

Results were classified into the following groups:

#### Class 1: emergency

Results that require the patient to apply to the emergency department immediately (intracranial bleeding, stroke, shift, skull fracture, cerebral venous sinus thrombosis, etc.).

#### Class 2: not emergency but needs timely attention

Reports in which the patient does not need to apply to the emergency room but requires outpatient clinic service in a health institution as soon as possible (intracranial space-occupying lesion, change of lesions during follow-up, metabolic diseases, etc.).

#### Class 3: clinically Non-significant

Reports in which the patient does not need an outpatient clinical service as soon as possible; the primary physician can read the report anytime (arachnoid cyst, age-related changes, calcifications, variations, etc.).

#### Class 4: normal

Completely normal findings.

Brain CT images between November 1, 2023, and June 1, 2024 were anonymized and transmitted to three tertiary referral hospitals: two public and one private. The patient's age, gender, comorbidites and other sociodemographic findings were among the electronically gathered referral data. We noticed 139 reports included mixed cases other than those relevant to brain CTs. Such cases are not in the scope of this study and, therefore, have been excluded from the dataset, leaving us 1,722 reports. Referrals that were incomplete or duplicated. Two radiologists with 2.5 years of experience classified and annotated the cases and all cases were reviewed by a board-certified radiologist with 7 years of experience. To ascertain the quality of our dataset, we curated pertinent reports under the study's objectives, thereby achieving a balanced dataset. We had the assumption that patients with multiple reports would usually have their first report as an emergency. To augment the quantity of emergency reports, we devised novel attributes for the reports, including the number of reports per patient, the character count of the report, and the approximate date. Subsequently, we employed a self-hosted Label Studio (v1.9.0) platform for collaborative data annotation by experts. Following this, we meticulously cleaned and aligned annotations with patient reports, yielding a well-prepared dataset for subsequent machine learning pipelines. This refined dataset provides a robust foundation for developing a precise and clinically significant medical AI model.

In the data ingestion phase, we utilized SQLalchemy (v2.0.33) to import the patient data into a PostgreSQL database. We indexed and anonymized sensitive patient data using a Fernet Key. All subsequent data transformations were executed within the database via dbt (v1.6.1), an extract-load-Transform (ELT) tool. Additionally, we leveraged the GPT 3.5 API to facilitate the translation of reports from Turkish to English. In order to ensure the precision of the translations, a subset of the reports was manually revised by 2 bilingual medical professionals fluent in Turkish and English language. The reviewers assessed the translations for precision, uniformity, and preservation of clinical sense. The prompt we utilized for this purpose was: Perform the following transformation on the report: “Translate into English”.

These steps align with the principles and practices recommended in the medical AI field, where high-quality data is of paramount importance for driving breakthroughs and advancing healthcare and medicine. Using a self-hosted Label Studio platform for collaborative data annotation is becoming a common practice in medical AI research and is widely preferred by experts. Therefore, experts could not only manage their annotation cycle but also prepare better-quality datasets. Furthermore, leveraging SQLalchemy and dbt for data ingestion and transformation is a standard procedure in many companies with large databases.

### Model architecture

**Figure 3:**
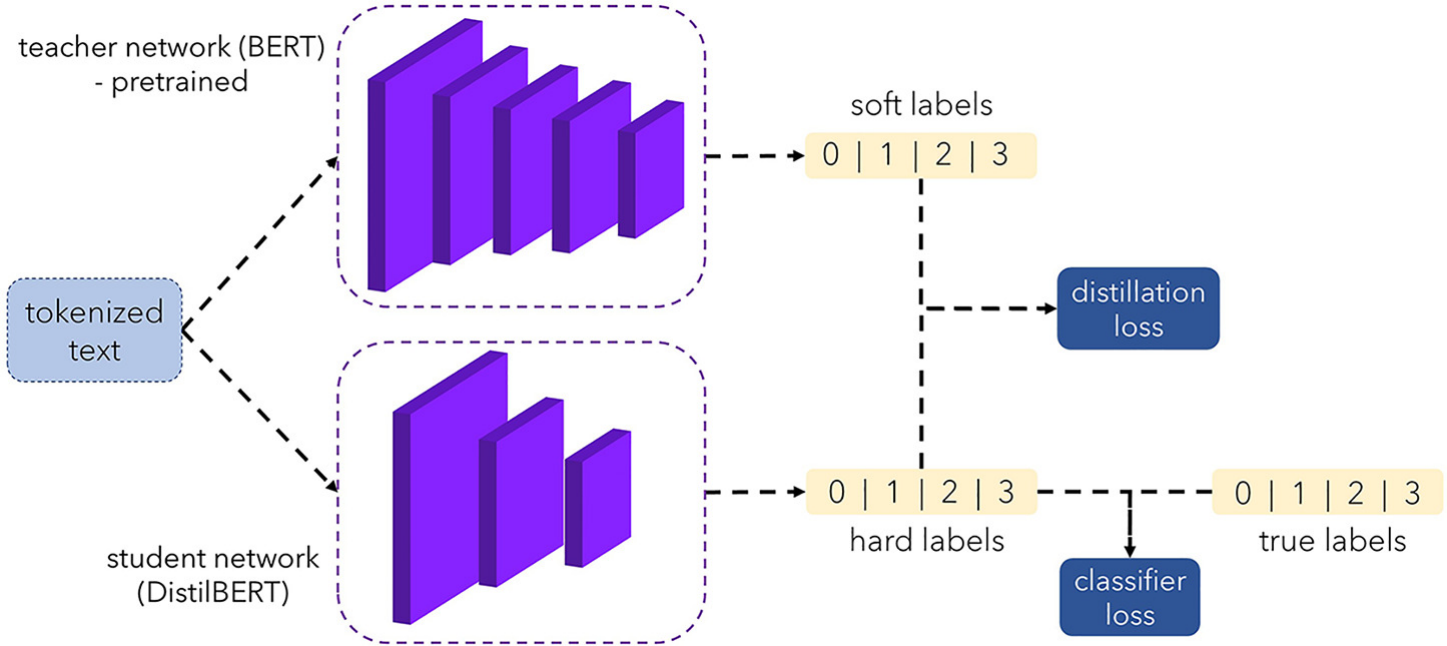
Accuracy folds and F1scores.

We are engrossed in categorizing text-based radiology reports into the four predefined classes. The task includes text classification of radiology reports rather than direct image classification. To implement our text classification approach on the prepared emergency room dataset, we adapted a large language model DistilBERT, a version of BERT with six transformer layers, 768 concealed units, and twelve attention heads. Researchers often use it for text organization by adding a fully connected output layer with soft-max activation to predict the urgency category. It contains around 66 million trainable parameters, in addition to the transformer layers and a fully coupled output layer. DistilBERT is proven to achieve efficiency without sacrificing accuracy (12). This lightweight model, compared to its counterpart BERT (13), is trained with a knowledge distillation technique to enable the same information to be learned with much fewer learnable parameters (14). Knowledge distillation, also known as the teacher-student framework incorporating two models, student network and teacher network, respectively. As shown in Figure 3, in knowledge distillation, DistilBERT is the student network that learns information from BERT as the teacher and becomes a smaller model (DistilBERT) that repeats the teacher's behavior (12, 15). The BERT (teacher) is already pre-trained; then, we train Distil BERT (student) learning to make predictions in a different context than the teacher. Moreover, we benefit from this feature, since our inputs and expected predictions have a medical context. We first tokenize (i.e., splitting the text into substrings containing multiple words) the text data by using the Distil BERT tokenizer with sequences amplified to a maximum length of up to 512 tokens. Next, we train a text classifier model with the Distil BERT backbone and then evaluate the classification results. All the data was split into three and tested with five-fold cross-validation: 689 reports for training, 172 for validation, and 861 reports reserved for testing (i.e., with a split ratio of 4:1:5). The trained classifier then tested on the remaining 861 annotation reports.

The full labeling methodology, model training pipeline, and evaluation metrics are available in our public GitHub repository (16, 17).

### Evaluation and results

**Figure 1:**
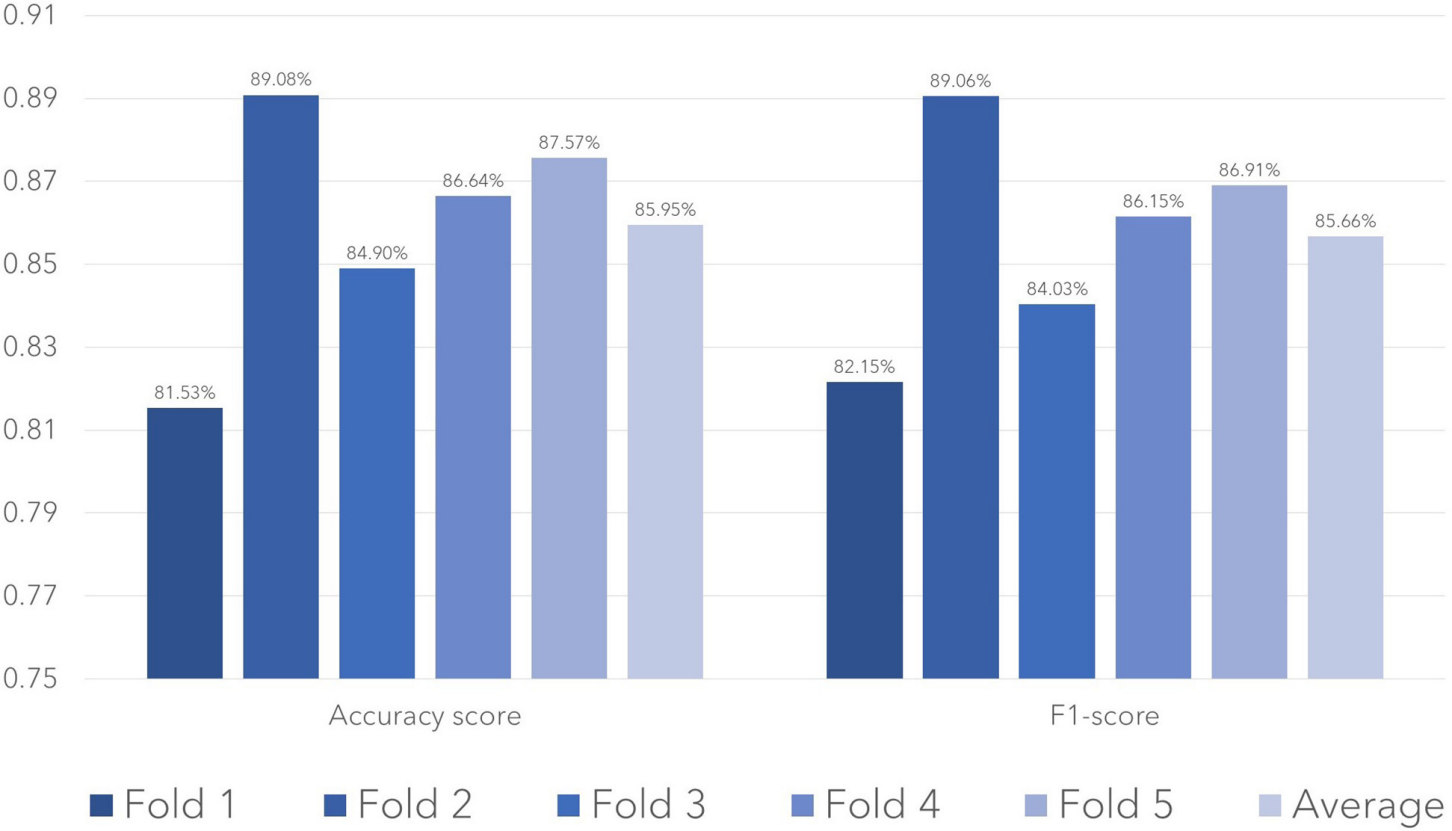
Test performance in terms of accuracy and F1-score for the models trained in each fold.

**Figure 2:**
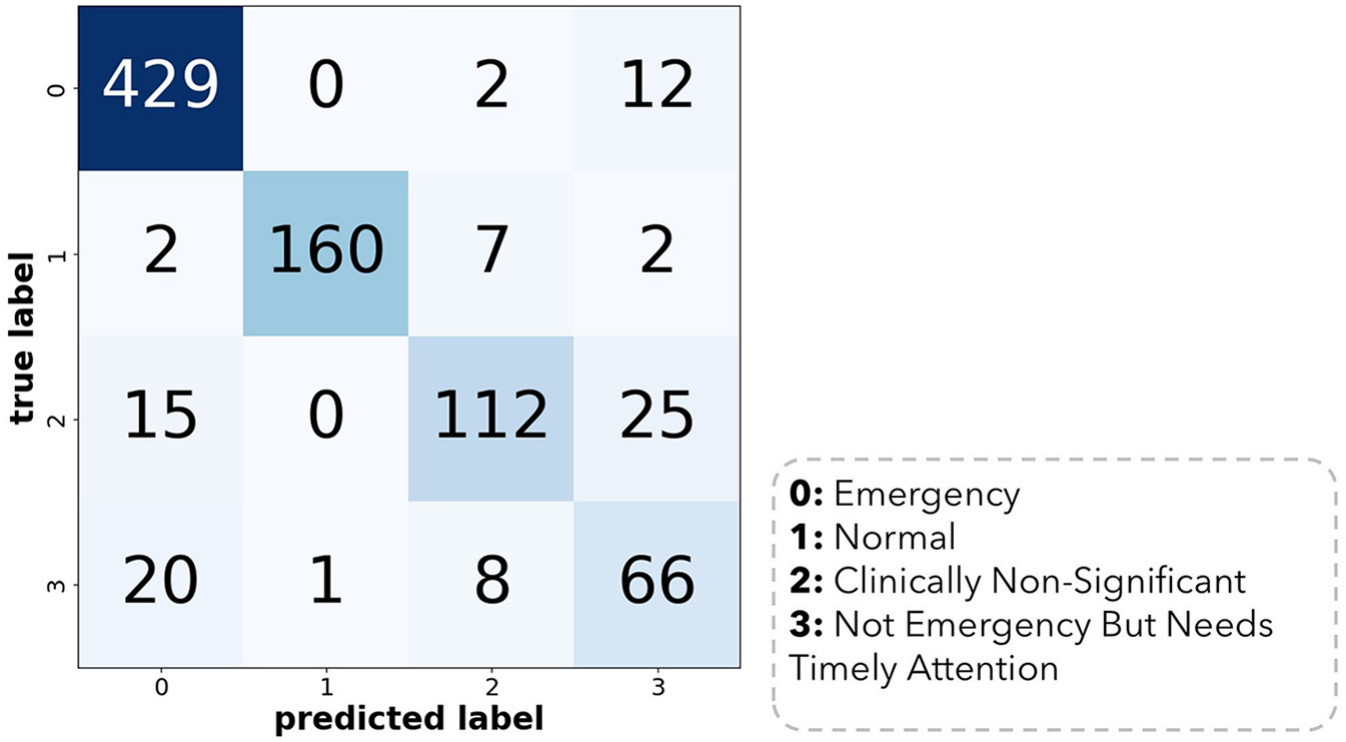
Confusion matrix of the best model trained across 5 cross-validation folds.

We first evaluate the representativeness (i.e., data coverage) of our prepared dataset. As per sociodemographic data, most of the participants had age range 19–35 years making 30% of the total population, with male subjects dominating the percentage (58%). Majority of the patients (40%) had no comorbidities and were mostly (80%) of Turkish nationality as tabulated in Table 1. To train the Distil BERT text classifier, we use the Adam optimizer with a learning rate of 2e-5 and a batch size of 32 alongside a cross-entropy loss function. A dropout rate of 0.1 was used to avoid overfitting. The training was made for 10 epochs, with early stopping being based on the validation loss. The training was processed on NVIDIA V-100 GPU, and it took about 4 h to complete. The model performance was assessed using accuracy, F1 score, and recall features. We performed five-fold cross-validation with a split ratio of 4:1:5 for training, validation, and testing, respectively. The trained classifier attained a mean F1 score of 0.85 across the entire folds, as shown in Figure 1. Out of 861 testing samples, 702 were correctly predicted with an overall accuracy of 81.5%. Class 3 has the highest misclassification rate. The confusion matrix for different classes is provided in Figure 2. We observe that the algorithm produced has a sensitivity of 91% and a specificity of 90% in the measurements made on the test data. The F1 score was measured as 0.89 for the best fold residing within well-performing model F1 scores, i.e., 0.80–0.90. We also highlight that in the worst-case scenario, the model can predict decently with approximately 0.81 F1 score. The best-performing model across the folds most successfully distinguished emergency results. However, in medical practice, false-negatives for the emergency class should be strictly avoided. Therefore, the model with the least false negative emergency cases should be preferred. The average recall value of 0.89 (moderate recall, 0.70–0.90) indicates that our model is able to indicate most of the actual positive instances, but a moderate number of false negatives indicates a better performance of the model as shown in Figure 1.

**Table 1:** Sociodemographic of the participant.

Variable	Feature	Percentage (%)
Age		
	Birth-18 years	05
	19–35	30
	36–50	25
	51–65	20
	>65	20
Gender		
	Male	58
	Female	42
Comorbidities		
	None	40
	HTN	20
	DM	15
	CVD	10
	Respiratory diseases	10
	Others	05
Ethnicity		
	Turkish	80
	Others	20

CVD, cardiovascular disease; DM, diabetes mellitus; HTN, hypertension.

### Retrospective justification audit

A total of 1,861 referrals were initially collected after the annotation; all the data was split in two; the first 1,000 (53.7%) reports were divided, with 800 (42.9%) used for training and 200 (10.7%) used for validation. The DistilBERT model was used for training. The resulting algorithm was tested on the remaining 861 annotated reports, as given in figure one below.

### Prediction of accuracy

Out of 861, 702 were correctly predicted with an overall accuracy of 81.5%. Class 3 has the highest misclassification rate. The confusion matrix for the best model is provided in Figure 2 below.

Provided below are the expected vs. predicted plot of class 0 in Table 2:

**Table 2:** Expected vs. Predicted Plot of Class 0.

Class 0	Expected	
Predicted	372 (+Ve)	67 (-Ve)
	21 (-Ve)	403 (+Ve)

It was observed that the algorithm produced sensitivity of 91% and a specificity of 90% in the measurements made on the test data. The F1 score was measured as 0.89 for the best fold. The algorithm most successfully distinguished emergency results. In order to evaluate the performance of the model, we performed K-fold cross-validation, where it was observed that with an average of 0.85 F1 score, residing within well-performing model values, i.e., 0.80–0.90. The accuracy folds along with the F1 scores are given in Figure 3 below:

The average recall value of 0.89 (moderate recall, 0.70–0.90) indicates that our model is able to indicate most of the actual positive instances, but a moderate number of false negatives indicate a better performance of the model, as given in the Table 3 below.

**Table 3:** Accuracy, recall, and F1 scores of folds.

Folds	Accuracy	Recall	F 1 score
1	0.82	0.85	0.82
2	0.89	0.93	0.89
3	0.85	0.87	0.84
4	0.87	0.90	0.86
5	0.88	0.91	087
Mean of all folds	0.86	0.89	0.85

## Discussion

At our clinical site, we conducted a retrospective analysis to evaluate the performance of a deep learning-based decision support system (DSS) in identifying brain MRIs without contrast. We assessed the diagnostic accuracy of self-hosted Label Studio and identified common causes of false-positive results. We found that the algorithm was able to classify the reports successfully with high performance, similar to previous studies (18, 19). Our findings indicate that mostly artifacts in the report are associated with reduced algorithm performance. Furthermore, we demonstrate that diagnostic effectiveness is independent of quantitative variations in image quality when evaluated by CT textural studies. Our study demonstrates the effectiveness of AI-based report assessment for predicting clinical reports. With a sensitivity of 91% and a specificity of 90%, the algorithm yielded an overall accuracy of 81.5%. The K-fold cross-validation results in our study indicated that the model performed well across all folds, with an average F1 score of 0.85 and an average recall value of 0.89.

With an average of 0.85 F1 score and recall value of 0.89, we have found a small number of false-positives that are directly attributable to various apparatuses used for CT scans. These examples, however, do not entirely explain why postsurgical patients perform worse. We cannot conclusively determine the mechanisms underlying this observation because neural networks' mechanisms are not easily examined; however, given these changes in diagnostic performance, we hypothesized that the effectiveness of AI tools may also depend on the characteristics of images. Future deep learning models for the classification may complicate or prevent interpretation by human readers.

We then investigated whether the false-negative results were due to poor image quality. Poor image quality can impact reporting, as such images are challenging for radiologists to interpret. Regardless of image quality, classifying hundreds of cases by urgency during daily practice can be difficult or even impossible for human experts. Therefore, we hypothesized that misclassification could similarly influence the performance of AI systems. In particular, discrepancies in diagnostic performance may result from subpar picture quality or from notable variations in image quality; also, studies that were mistakenly flagged may have had lower-quality images than studies that were successfully flagged.

As examples, beam hardening artifacts (20) and excessive noise (21) change the textural properties of CT data sets and the reports. Since the reports of these artifacts are vague, their classification can be difficult for the AI system.

## Conclusions

In this study, we investigated the diagnostic precision of a deep learning algorithm for classifying the non-contrast Brain CT reports. Our results demonstrate that the proposed algorithm achieves high accuracy, sensitivity, and specificity in classifying non-contrast brain CT scan reports. The algorithm's performance was evaluated on a dataset of 861 CT scan reports, which were annotated by expert radiologists to establish a gold standard.

Our findings highlight the feasibility of automating critical case identification, which could enhance workflow efficiency and expedite patient management in high-volume settings. By accurately distinguishing urgent from non-urgent cases, our approach may contribute to reducing reporting delays and optimizing resource allocation. Future studies should explore the clinical impact of this classification system and its integration into real-world radiology workflows.

## Data Availability

The raw data supporting the conclusions of this article will be made available by the authors, without undue reservation.
