# Correction: Diagnostic precision of a deep learning algorithm for the classification of non-contrast brain CT reports

**DOI:** 10.3389/fradi.2025.1744006

**Published:** 2025-11-24

**Authors:** Hamza Eren Güzel, Göktuğ Aşcı, Oytun Demirbilek, Tuğçe Doğa Özdemir, Pelin Berfin Erekli

**Affiliations:** 1 Department of Radiology, İzmir City Hospital, İzmir, Türkiye; 2 School of Science and Technology, IE University, Segovia, Spain; 3 School of Management, Technical University of Munich, Munich, Germany

**Keywords:** artificial intelligence, report classification, computed tomography, deep learning, noncontrast head CT

In the published article, there was a mistake in the conclusion part of the abstract:

This study revealed decreased diagnostic accuracy of an AI decision support system (DSS) at our institution. Despite extensive evaluation, we were unable to identify the source of this discrepancy, raising concerns about the generalizability of these tools with indeterminate failure modes. These results further highlight the need for standardized study design to allow for rigorous and reproducible site-to-site comparison of emerging deep learning technologies.

This has been corrected to read:

The proposed deep learning algorithm demonstrated high diagnostic accuracy, sensitivity, and specificity in classifying non-contrast brain CT reports. These results indicate the feasibility of automated identification of critical cases, which may support workflow efficiency and timely patient management in radiology practice.

The original version of this article has been updated.

